# Local COVID-19 Severity and Social Media Responses: Evidence From China

**DOI:** 10.1109/ACCESS.2020.3037248

**Published:** 2020-11-10

**Authors:** Ting Da, Liang Yang

**Affiliations:** Xi’an Microelectronics Technology Institute Xi’an 710065 China

**Keywords:** COVID-19, linear probit, random forest, Sina Weibo, sentiments

## Abstract

Unexpected but exceedingly consequential, the COVID-19 outbreak has undermined livelihoods, disrupted the economy, induced upheavals, and posed challenges to government decision-makers. Under various behavioural regulations, such as social distancing and transport limitations, social media has become the central platform on which people from all regions, regardless of local COVID-19 severity, share their feelings and exchange thoughts. Our study illustrates the evolution of moods expressed on social media regarding COVID-19-related issues and empirically confirms the hypothesis that the severity of the pandemic substantially correlates with these sentiments by analysing tweets on Sina Weibo (China’s central social media platform). Methodologically, we leveraged Sentiment Knowledge Enhanced Pre-training, the most state-of-the-art natural language processing pre-trained sentiment-related multipurpose model, to label Sina Weibo tweets during the most distressed period in 2020. Given that the model itself does not provide a feature explanation, we utilize a random forest and linear probit model with the labelled sample to demonstrate how each word plays a role in the prediction. Finally, we demonstrate a strong negative linear relationship between the local severity of COVID-19 and the local sentiment response by incorporating miscellaneous geo-economic control variables. In short, our study reveals how pandemics affect local sentiment and, in a broader sense, provides an easy-to-implement and explanatory pipeline to classify sentiments and resolve related socioeconomic issues.

## I. Introduction

Persistent and consequential, COVID-19 has resulted in a sequence of serious social and economic problems worldwide. With the social distancing measures (such as community quarantines and store closures) to counter the spread of the virus, individuals have been forced to express their opinions and thoughts on social media, such as Twitter in the US and Sina Weibo in China. Concerning the potential bidirectional causality between the fear engendered by the pandemic and socioeconomic upheavals, for instance, [1], it is therefore crucial for policymakers to empirically investigate the relationship between social sentiments and COVID-19 severity and to implement policies accordingly.

To statistically examine how the pandemic has shaped sentiment changes, we propose a three-stage model that sequentially: (i) labels Sina Weibo tweets using the state-of-the-art pre-trained sentiment model Sentiment Knowledge Enhanced pre-training (SKEP) [2]; (ii) illustrates individual word influence through a random forest (RF) and linear probit (LP) model; and finally (iii) establishes the inverse relationship through linear regression.

In the first stage, a sentiment analysis, in practice, primarily addresses user reviews (e.g., [3]–[8]) and social media texts (e.g., [9]–[14]). Major downstream tasks involve sentiment polarity classification at the sentence or aspect level and opinion extraction, among others. Conventionally, researchers have separately built specific models for these tasks; the basis is either artificially designed features [15] or neural networks [11]–[14], [16].

Recently, the natural language processing community has witnessed significant breakthroughs in pre-trained methods for capturing general semantic representations at the word level [17]–[19]. The learning objectives of these studies usually have masked language modelling [19] or next-word prediction [18], and pre-trained results can serve as the foundation for a variety of downstream sentiment analysis tasks. For example, the Bidirectional Encoder Representations from Transformers (BERT) model [19] has been applied to categorise randomly selected Sina Weibo tweets in an unsupervised manner [21].

Aside from pre-trained models, a cohort of methods has been proposed to classify sentiments from scratch. Speaking of exploratory analysis, WordCloud was used to trace the dominant public mood during the peak period of the pandemic in India during late March, 2020 [22]. On a broader time scale, a WorldCloud of more than 20 million Twitter tweets filtered by COVID-19-related keywords demonstrated that, since the outbreak in January 2020, anger and joy were gradually catching up to the dominant mood – fear – towards the end of April, 2020 [23].

In terms of technical classification, for instance, the performance of a collection of classifiers, such as decision tree, random forest (RF), and support vector machine (SVM), for labelling sentiments of COVID-specific Twitter tweets, was evaluated [24]. Another implementation of classical machine learning methods, Naive Bayes and logistic regression, focuses on not only assigning numerical sentiment scores, but also classifying tweet categories into fear, sadness, and anger. It turns out that both algorithms achieve decent prediction accuracy, particularly when tweets are short [25]. For another instance, a pipeline consisting of the classic BiLSTM + attention + CRF model was applied to simultaneously label COVID-related sentiments and extract emotional words [26]. In other applications, a recurrent neural network (RNN) incorporating details of the topic themes was also implemented to zoom in on predicting the deviation of emotional polarity from being neutral, and it obtained finer sentiment ordinals than the established popular Python package, “TextBlob” [27].

In our study, we adopted SKEP as the sentiment classification model since it builds upon previous pre-training tricks (particularly [19], [20]) and integrates three key pieces of sentiment knowledge, namely, sentiment words, word polarity, and aspect-sentiment pairs, into pre-training and therefore produces more informative representations specific to sentiment-related tasks. Moreover, various SKEP experiments have confirmed its superiority over most of the leading pre-trained models.

In addition to technical studies on acquiring the accurate classification of COVID-19 sentiments, several studies have been dedicated to establishing the causality between moods and real socioeconomic variables during the pandemic. For instance, examining firm-level labelled public opinions (e.g., news articles) from Truvalue Labs’s data on environmental, social, and governance (ESG), it was shown that fewer negative stock returns are usually accompanied by more positive public sentiment towards a company’s response (e.g., measures to avoid large-scale layoffs) [28]. The socioeconomic factors driving an individual’s sentiments on Twitter about reopening the economy has also been investigated [29]. Regarding China’s financial markets, a time-series sentiment index was constructed using SVM based on China’s official news media, as well as Weibo; the results showed how sentiment could amplify a pandemic-induced economic crisis by a positive relationship between COVID-19 sentiments and stock returns [30]. From a global perspective, cross-country text media sentiment was analysed using a panel regression, concluding that the US stock market responds more sensitively to sentiment than confirmed cases [31].

In short, our contributions are fourfold.

First, we utilize the most advanced pre-trained sentiment model, SKEP, to classify tweets on social media, which not only runs fast but also produces sensible predictions.

Second, because SKEP, as a neural network, does not report how each feature (i.e., word) plays a role in assigning labels, we further provide feature explanations (importance and sign) using an RF and LP model.

Third, by aggregating tweets and labels at the province level, we demonstrate a strong, inverse linear relationship between the local COVID-19 confirmed cases and the mean sentiment responses. The results empirically confirm the common hypothesis that a more severely affected area will experience a significant decrease in sentiment polarity towards pessimism.

Fourth, our pipeline balances accuracy, interpretability, and convenience, while existing approaches have either entailed time- and resource-consuming neural network training specific to the problem for decent performance (but lost interpretability) or sacrificed accuracy for convenience by utilizing traditional general-purpose machine learning algorithms.

## II. Framework

**FIGURE 1.**
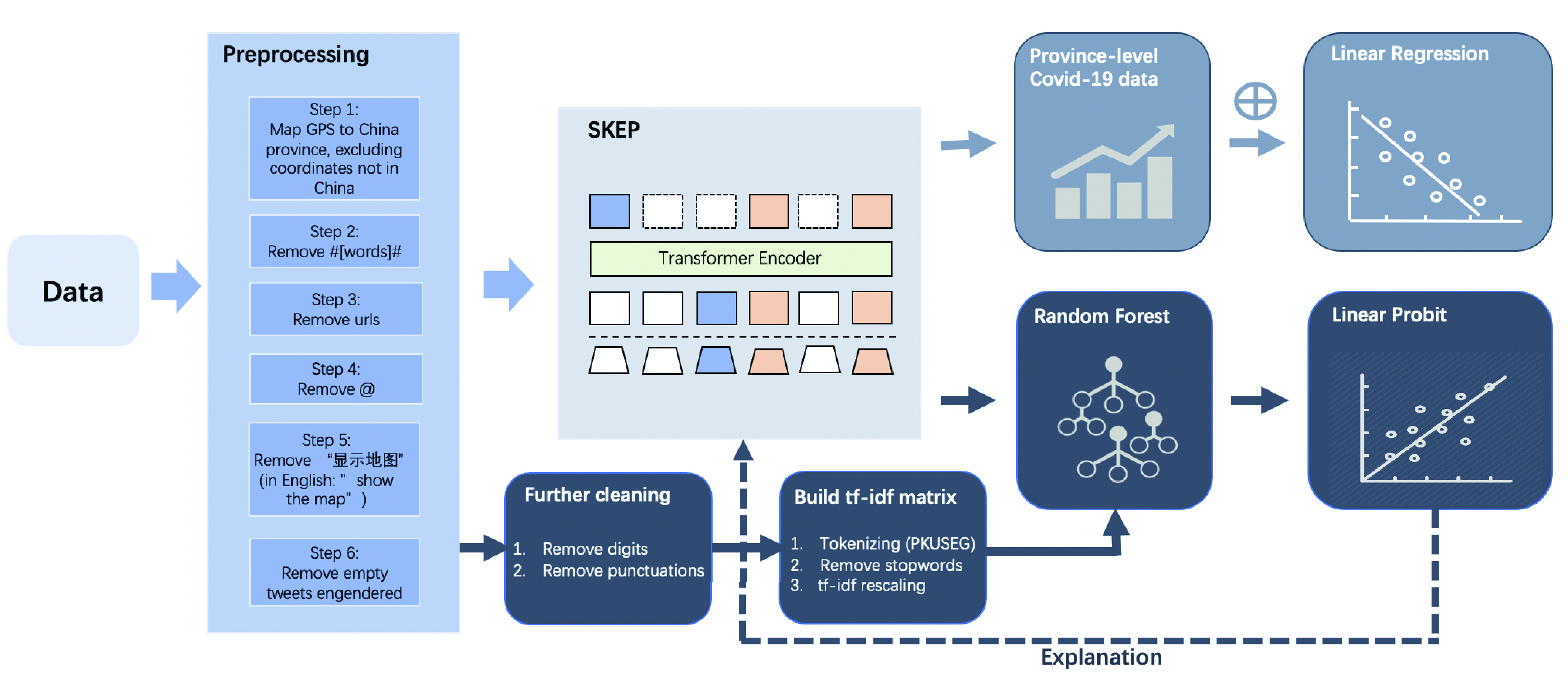
Three-stage model pipeline.

As illustrated in Fig. 1, the model pipeline consists of a preprocessing step followed by SKEP, a pre-trained model for classifying Sina Weibo’s tweet sentiments and further analyses. Two sets of studies were conducted after SKEP. First, coupled with China’s provincial COVID-19 confirmed case data from February 2020, we implemented a linear regression to demonstrate causality from the local COVID-19 severity to sentiment drop, by accounting for various socioeconomic endogeneity concerns and heterogeneity across provinces.

Second, because the pre-trained SKEP model is, by nature, a neural network, the model itself does not provide a feature explanation. To shed light on how each word plays a role in driving the prediction, we performed an RF to select influential words, and we ran an LP model to capture the sign of each word. Overall, the motivation here was to open the black box of SKEP and confirm the validity of the SKEP classification through an RF and LP.

**FIGURE 2.**
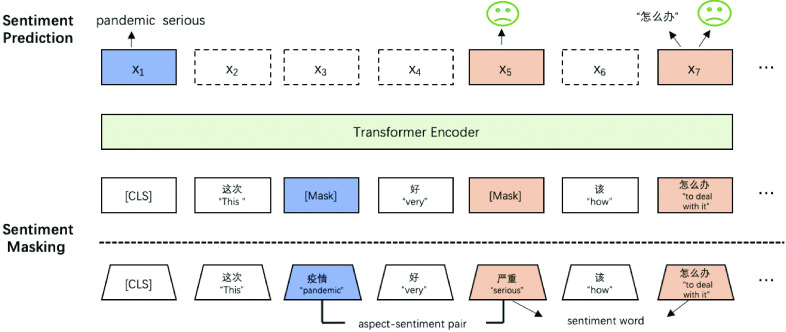
SKEP structure.

Fig. 2 demonstrates masking procedure and the joint optimisation. Note that the English words in quotation marks are translations of the original Chinese words above them; in our analysis, we work directly with tokenised Chinese words, not their translation. In short, masking corrupts the input sequence in light of $\mathcal {K}$, and the pre-training objectives are to maximally recover the masked information through a transformer. The three pieces of objectives are: (i) sentiment word prediction ($x_{7}$); (ii) word polarity classification ($x_{5}$ and $x_{7}$); and (iii) aspect-sentiment pair prediction ($x_{1}$). Note also that the algorithm does not predict the sentiment word on $x_{5}$, because it has already been predicted on $x_{1}$.

We explain the details of each stage separately as follows.

### A. Stage 1 – Sentiment Knowledge Enhanced Pre-Training

While conventional sentiment analyses are prone to separately examining different types of sentiment knowledge for diverse downstream applications, SKEP simultaneously learns three types of sentiment knowledge (i.e., sentiment words, word polarity, and aspect-sentiment pairs). Such a joint training process endows the model with greater versatility than assorted sentiment analysis tasks.

Considering the training procedure, SKEP first conducts an automatic mining of sentiment knowledge $\mathcal {K}$ from the unlabelled input text sequence $X$ (Section II-A1). Guided by $\mathcal {K}$, the algorithm then masks a portion of texts to generate a corrupted version of training data $X'$ (Section II-A2). $(X,X')$ are actually parallel data. Next, SKEP uses a transformer to recover sentiment information for $X'$ (Section II-A3).

Eventually, for a given input sentence, the ultimate state vector of [CLS], the classification token, is considered the overall representation, from which the sentiment probability is calculated by inserting a classification layer on top of the transformer encoder.

We discuss steps 1 to 3 in greater detail in the following subsections.

#### 1) Sentiment Knowledge Mining

SKEP mines sentiment knowledge based on a simple and intuitive approach, i.e., point mutual information (PMI) [32], which is widely used in information retrieval. PMI generally indicates whether one sees a pair of words together more often than if they are independently. The PMI score is calculated by

\begin{equation*} PMI(x_{1},x_{2}) = \log \dfrac {p(x_{1},x_{2})}{p(x_{1})\cdot p(x_{2})},\tag{1}\end{equation*}

where $p(\cdot)$ is the probability estimated by counts.

For the purpose of obtaining sentiment knowledge, the model is interested in only the word pairs that involve at least a sentiment seed word $\tilde {s}$. In the pre-trained version, the set of seed words contains 25 positive and 21 negative words. The algorithm then computes a word polarity score for a candidate word $x^{*}$, $WP(x^{*})$ as

\begin{equation*} WP(x^{*}) = \sum _{WP(\tilde {s})=+} PMI(x^{*},\tilde {s}) - \sum _{WP(\tilde {s})=-} PMI(x^{*},\tilde {s}).\tag{2}\end{equation*}

In other words, the word polarity is computed by calculating the difference between its PMI scores with all of the positive seeds against all of the negative ones. A positive $WP(x^{*})$ means that $x^{*}$ is a positive word and vice versa. After obtaining sentiment words, SKEP extracts aspect-sentiment pairs defined by a sentiment word with its closest word, which is a noun. The distance between the centered sentiment word and the candidate noun is empirically set to be at most three tokens at most. Sentiment words and their aspect-sentiment pairs are then collected as mined sentiment knowledge $\mathcal {K}$.

#### 2) Sentiment Masking

Inspired by BERT, which first proposed the masked language modelling objective to pre-train the transformer encoder and achieved tremendous improvement of multifarious downstream tasks, SKEP also introduces a masking process to create a noisy version of the original text sequence.

However, SKEP differs from BERT in the units to be masked and how the units are selected. In BERT, the unit is only a token. In SKEP, however, the units are sentiment words and aspect-sentiment pairs gathered by $\mathcal {K}$ and likely other tokens. Moreover, by treating each token equally, BERT uniformly selects the items to be masked. In contrast, SKEP sequentially performs the following process: First, the algorithm randomly selects at most two aspect-sentiment pairs in a sequence and replaces each token in them by [MASK]. Second, a subset of the remaining unmasked sentiment words is masked, but the total number of tokens masked up until this step could not exceed 10%. Finally, provided insufficient masked tokens (e.g., less than 10%), SKEP would meet the number constraint on the total masked tokens by replacing some common tokens by [MASK].

As illustrated in Fig. 2, “pandemic” and “serious” are substituted by [MASK] because they are aspect-sentiment pairs, and “to deal with” becomes [MASK] because it is a sentiment word.

#### 3) Sentiment Pre-Training Optimisation

Given the corrupted data $X'$ after the masking procedure, the transformer encoder is then asked to recover the masked sentiment information. Accordingly, the algorithm optimises against the objective defined as the sum of the three types of losses:

\begin{equation*} loss_{total} = loss_{sw} + loss_{wp} + loss_{ap},\tag{3}\end{equation*}

where $loss_{sw}$, $loss_{wp}$, and $loss_{ap}$ are, respectively, the objectives for the three sorts of masks created in Step 2 – sentiment word, word polarity, and aspect-sentiment pairs.

Technically, $loss_{sw}$ is defined to maximise the probability of recovering the original sentiment word based on the transformer encoder. The purpose here is remarkably different from that in BERT, which popularised such a masking tricks. In BERT, masked words were first randomly selected, but SKEP restricts the masked words to those that are sentiment-related. Thus, SKEP is more appropriate for sentiment tasks.

In addition, considering the masked sentiment token, $loss_{wp}$ gauges the difference between the word polarity (predicted by the output of the transformer encoder) and the polarity of the original sentiment word (obtained in $\mathcal {K}$). In addition, $loss_{ap}$ measures how our prediction of the masked aspect-sentiment pairs is different from the original version. The ultimate state of the classification token [CLS] (the representation of the entire text sequence) is used here to predict the pairs.

### B. Stage 2: RF and LP

The central idea is that, assuming that SKEP makes reasonable predictions about tweets, then what are the important features (i.e., words) considered by the model, and do they satisfy our common sense? Because SKEP itself does not offer a feature importance score, we turn to an RF that not only enjoys sound in-sample and out-of-sample performances but also measures feature contribution. We also employ an LP model with the most important features selected by the RF to assign a coefficient to each word, indicating whether the occurrence of the word will drive the algorithm to label it as 1 or 0.

#### 1) RF Input

In our study, the outcome is a binary variable, indicating whether a piece of tweet text is a positive (i.e., 1) or negative (i.e., 0) sentiment labelled by SKEP. The input is the classical term-frequency-inverse-document-frequency (tf-idf) matrix, $W$, with documents (i.e., tweets) in the rows and distinct words in the columns. In other words, $W_{ij}$, the $(i,j)$th entry in $W$, is the number of times the $j$th word in vocabulary is shown in the $i$th document scaled by a factor. In tf-idf, in any column $j$, the factor is chosen as

\begin{equation*} \log \left({\dfrac {\text {Total number of documents}}{\text {Number of documents containing word } j}}\right),\end{equation*}

where frequent words effectively have low weight. The procedure helps to reduce the noise engendered by meaningless stop words and to condense the feature space.

In the experiment, we performed two further cleaning steps (remove digits and punctuation) prior to the tf-idf transformation. These steps are widely used in text preprocessing, because the goal here is to focus only on how meaningful words will play parts in classifying sentiments, not digits or punctuations. In particular, these steps were not included in Stage 1 because the pre-trained SKEP model could automatically address numbers and punctuations without any interference.

Moreover, for simplicity and interpretation concerns, we considered only $1-gram$ of words, and the tokenisation was performed using a particular version of PKUSEG [33], an algorithm for Chinese word segmentation that specializes with social media texts. After further removing the stop words (in total, 1,396 commonly used English and Chinese stop words), we obtain a set of features that only consist of a single word in the corpus, instead of consecutive words.

#### 2) RF Mechanism

RF is a multipurpose algorithm that can be used for classification and regression tasks; it grows a collection of decision trees and makes predictions based on voting systems. In practice, RF selects a bootstrap sample of tweets and feeds them into an empty decision tree. At each node of the tree, the algorithm chooses the feature that could best improve impurity, a measure of how “pure” the split of the sample is after carving out the observations at the optimised threshold of this particular feature. The process is repeated for many times to obtain a “forest” (i.e., collection) of trees. In the last stage, the algorithm asks each tree to make a classification and sets the winner of the majority vote as the final prediction of the input tweet.

In particular, the trick that empowers RF to achieve a decent out-of-sample prediction accuracy and to train faster than the bagging of trees is that, at each split decision, the tree is only allowed to consider a subset of all features [34]. We use the convention in our experiment that the subset contains $\sqrt {p}$ predictors, where $p$ is the total number of features. The rationale is that, in the presence of a set of strong features, most trees will very likely choose one of these strong predictors in their initial splits, resulting in a similar tree structure at the end. Voting based on these highly correlated trees would not achieve as much reduction in variance as voting by uncorrelated trees. Thus, by ensuring that each tree looks only at a random subset of predictors, RF affords other predictors the opportunity to play a role in forming the split and therefore renders the trees uncorrelated.

#### 3) Feature Importance

Once the model is trained, we identify the features that play prominent roles in driving up the prediction performance. Similar to the node split process in the decision tree, in RF, the condition determining whether a particular feature should be chosen as the one to split observations is also based on impurity. Specifically, each decision tree in the RF selects the feature that could maximally reduce impurity at each round of node splitting. Thus, the contribution of each feature could be calculated by averaging the decrease in impurity induced by the feature across all trees.

Sorting feature importance from high to low, we retain a subset of all word features and feed them into the linear probit model. The goals are twofold. First, by maintaining a small number of features, we are able to interpret the words that are informative to sentiment classification. Second, by restricting to only, say, the top 50 features, we are able to pass them as the regressors in the linear probit model that will estimate the sign for each word, without worrying about the issue that the number of regressors exceeds observations.

#### 4) LP

The LP model is a special case of the cross-sectional linear regression model, as outlined in the next chapter. The model was established as follows:

\begin{equation*} \vec {Y} = X\vec {\beta } + \epsilon,\tag{4}\end{equation*}

We discuss various empirical concerns in the next section. The upshot is $E[\epsilon _{i}|X_{i}] = 0$, the central identifying condition in linear regression that ensures the unbiasedness, consistency, and asymptotic normality of the estimated $\vec {\beta }$, although the LP model will yield predicted values outside $\{0,1\}$, the set of $Y$.

In fact, we ask for less from the LP. We are only concerned with the predicted signs of each word. In other words, the of the estimated coefficients illustrate whether the occurrence of words, such as “ICU” or “shortage”, will drive the predicted sentiment label to 1 or 0. In this way, the results of the LP model serve as a supplement to the feature importance given by the RF (the latter has no sign). Furthermore, we implemented the LP model after the RF because we wanted to ensure that there would be fewer features than observations. Otherwise, the LP model would fail to provide coefficient estimates.

### C. Stage 3: Linear Regression

In the final step, we empirically confirm the causal relationship between COVID-19 and Weibo sentiment at the province level. The model is

\begin{equation*} Sent_{i} = \alpha + \beta \cdot \log (Cases_{i}) + \sum _{j} \gamma _{i}^{(j)}\cdot C_{i}^{(j)} + \epsilon _{i}, \tag{5}\end{equation*}

where $Sent_{i}$ is the proportion of positive sentiments in $province_{i}$, $\alpha $ is the intercept, $\log (Cases_{i})$ is the $\log $ of confirmed cases, and $C_{i}^{(j)}$’s are various control variables accounting for baseline provincial heterogeneity such as population, GDP per capita, and the share of manufacturing industry, among others.

Our baseline gauge of provincial exposure to COVID-19 is the locally confirmed cases. We prefer confirmed cases to deaths because the distribution of deaths is extremely skewed to the left with only one province, Wuhan, exceeding 50 and reaching 2,761 by the end of February 2020. In contrast, the number of confirmed cases is more smoothly distributed across provinces and has reasonable variation to obtain a low variance of estimated coefficients.

#### 1) Empirical Concerns

To ensure that (5) truly captures the impact of the local pandemic severity on sentiment responses, we consider three major endogeneity concerns: (i) omitted variable bias; (ii) simultaneity; and (iii) sample selection bias. Satisfying all of these conditions, the coefficient estimates truly capture the one-way causality from $Cases_{i}$ to $Sent_{i}$.

Considering the potential omitted variables, the main identifying assumption of (5) is that $\log (Cases_{i})$ is independent of other time-varying regional economic shocks, and $Sent_{i}$ is estimated from only Sina Weibo tweets that contain COVID-19-related keywords. Thus, $\beta $ should reveal only the influence of local pandemic severity, rather than other potentially omitted shocks in $\epsilon _{i}$.

In addition, considering simultaneity, there is no obvious reverse causation from $Sent_{i}$ to $\log (Cases_{i})$ because the growth of confirmed cases is primarily affected by government regulations based on epidemiological knowledge and is coordinated by provincial and central governments, rather than local sentiments. In this light, there is no potential simultaneity problem; and therefore, $\beta $ should reflect only the one-way causation of the pandemic on local sentiments.

In short, accounting for the omitted variable bias and bidirectional causality between the responses and the main regressor, model (5) views the outbreak of COVID-19 as an exogenous shock, and the coefficient of $\log (Cases_{i})$ should therefore measure the direct effect of the pandemic on social media sentiments.

#### 2) Model Training

To paraphrase the linear model in the context of machine learning, (5) can be written as

\begin{equation*} Y = X\cdot \vec {\beta } + \epsilon,\tag{6}\end{equation*}

where $Y$ is an $n$ by 1 vector containing sentiment responses by each province – in our case, $n=31$ since we have 31 provinces in China. $X$ is an $n$ by $p$ matrix where $p$ is the number of features. $\vec {\beta }$ is the unknown parameter vector to estimate and $\epsilon $ is the residual.

The classical loss function for linear regression is the mean squared error (MSE):

\begin{equation*} loss = ||Y - X\vec {\beta }||^{2} = (Y - X\vec {\beta })^{T}(Y-X\vec {\beta }), \tag{7}\end{equation*}

Instead of applying gradient descent to minimise MSE in (7), we note that there actually exists an closed-form solution. Indeed, expanding (7), we would obtain

\begin{align*} loss=&(Y - X\vec {\beta })^{T}(Y-X\vec {\beta }) \tag{8}\\=&Y^{T}Y - 2\vec {\beta }^{T}X^{T}y + \vec {\beta }^{T}X^{T}X\vec {\beta }\tag{9}\end{align*}

The partial derivative of the loss with respect to $\vec {\beta }$ is

\begin{equation*} \dfrac {\partial loss}{\partial \vec {\beta }} = -2X^{T}Y + 2X^{T}X\vec {\beta }\tag{10}\end{equation*}

Setting the partial derivative to 0, we obtain the normal equation

\begin{equation*} X^{T}X\vec {\beta } = X^{T}Y\tag{11}\end{equation*}

Assuming that $X$ has full column rank, the optimal $\vec {\beta }$ is thus

\begin{equation*} \vec {\beta }^{*} = (X^{T}X)^{-1}X^{T}Y\tag{12}\end{equation*}

#### 3) Evaluating Model Performance

Unlike other mainstream machine learning algorithms that apply various analogies to MSE as the measure of goodness of fit, linear regression usually uses other techniques, such as $R^{2}$, to gauge model performance, because MSE has already been optimised globally by solving the first-order condition. In our model, we follow the convention of using $R^{2}$ because it offers a straightforward explanation for how a prediction is close to the true value.

$R^{2}$ consists of three components – all relevant to the sum of squares.

\begin{align*} SSTO=&\sum _{i=1}^{n} (y_{i} - \bar {y})^{2}\tag{13}\\ SSR=&\sum _{i=1}^{n} (\hat {y}_{i} - \bar {y})^{2}\tag{14}\\ SSE=&\sum _{i=1}^{n} (y_{i} - \hat {y}_{i})^{2}\tag{15}\end{align*}

In the formula, $\bar {y}$ is the mean of all responses, and $\hat {y}_{i}$’s are predictions. $SSTO$, $SSR$, and $SSE$ represent the total sum of squares, regression sum of squares, and error sum of squares, respectively. From this perspective, a good model should have a large proportion of $SSTO$ explained by $SSR$. That is, $R^{2} = \frac {SSR}{SSTO}$ should be relatively large.

## III. Empirical Units

### A. Data Overview

We focus on the empirical study on February 2020, the most distressed period in China during COVID-19, because the factors driving the decline in sentiments are less susceptible to noise (e.g., unawareness before the spike in case growth, post-February store reopening, and establishment of temporary hospitals), compared with the periods before and after February 2020. Such a restriction helps to clarify the causality from the local COVID-19 severity to the sentiment responses – the message to be conveyed in the 3rd stage.

Using data sources, our Sina Weibo tweet data were collected by [35]. The dataset has already been filtered (using a set of pre-defined COVID-19-related keywords) so that we are provided with only COVID-19-related Sina Weibo tweets. As shown in Table 1, there are a total of 10,815,385 public Sina Weibo tweets sent in February 2020. To determine the relationship between the local pandemic statistics and sentiment responses, we only considered tweets that contain GPS coordinate information, leaving us with 352,696 tweets. We further mapped these (latitude and longitude) pairs to the 31 provinces and special districts in China and obtained 343,528 observations; the decrease was mainly due to the GPS coordinates outside of China. In addition, the average length of Sina Weibo tweets decreased. One might wonder why the mean length was reduced to only 93.62 characters after restricting the sample to those containing GPS information. In fact, many of the deleted tweets are from official accounts with lengthy content and chained reposts with duplicated paragraphs. Typical types of these accounts are nationwide serious news reports, entertainment news, and doctors/hospitals giving advice about protecting people from the pandemic. In most cases, the GPS information is intentionally cloaked to show the professionalism of the official accounts.

**TABLE 1:** Basic Data Statistics

Statistics	All	Recognizable GEO	Cleaned for SKEP	Further cleaned
Total tweets	10,815,385	343,528	340,519	340,456
Mean length	190.43	93.62	76.50	66.85

We performed a series of cleaning steps in columns 3 to 4 of Table 1, as illustrated in Fig. 1. Sequentially, we removed tags, URLs, @’s, “show the map” (a string that always comes after GPS coordinates), and any empty observations engendered due to the cleaning. Thus, a total of 340,519 observations with an average length of 76.5 were obtained. The mean was large, more than 76 characters, because many emojis were recorded in their English names (e.g., “[good]”), increasing the counts of characters.

Moreover, from columns 4 to 5, we performed two more cleaning steps for RF: removing digits and punctuations. Finally, a total of 340,456 Sina Weibo tweets were obtained, but the average length further decreased to 66.85 characters.

**FIGURE 3.**
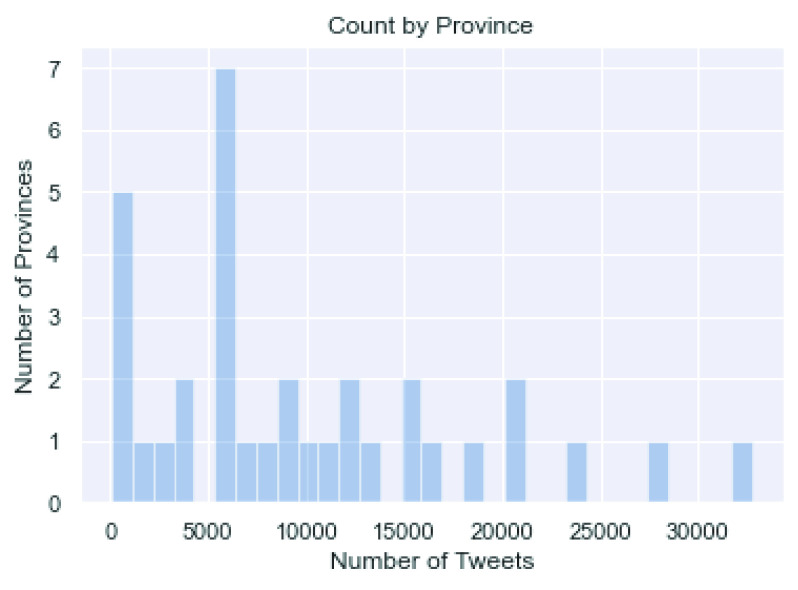
The count of Weibo tweets by province in February 2020.

**FIGURE 4.**
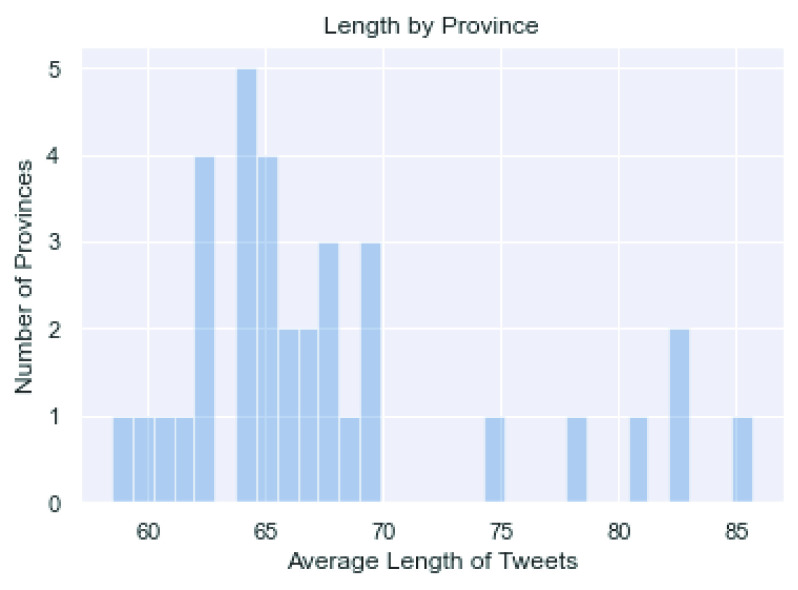
Mean length of Weibo tweets by province in February 2020.

Figs. 3 and 4 present the distribution of Sina Weibo tweets count and mean lengths across provinces, respectively. Both figures suggest that the range in either statistic is reasonable with no obvious and influential outliers. Particularly, Fig. 4 suggests that the lengths of tweets are comparable across provinces.

The other part of the input used in our empirical study is the province-level daily COVID-19 confirmed cases in China. The data come primarily from the National Health Commission of the People’s Republic of China and were formally collected by [36]. For the Sina Weibo data, we focus on the trend of COVID-19 confirmed cases on February 2020.

**FIGURE 5.**
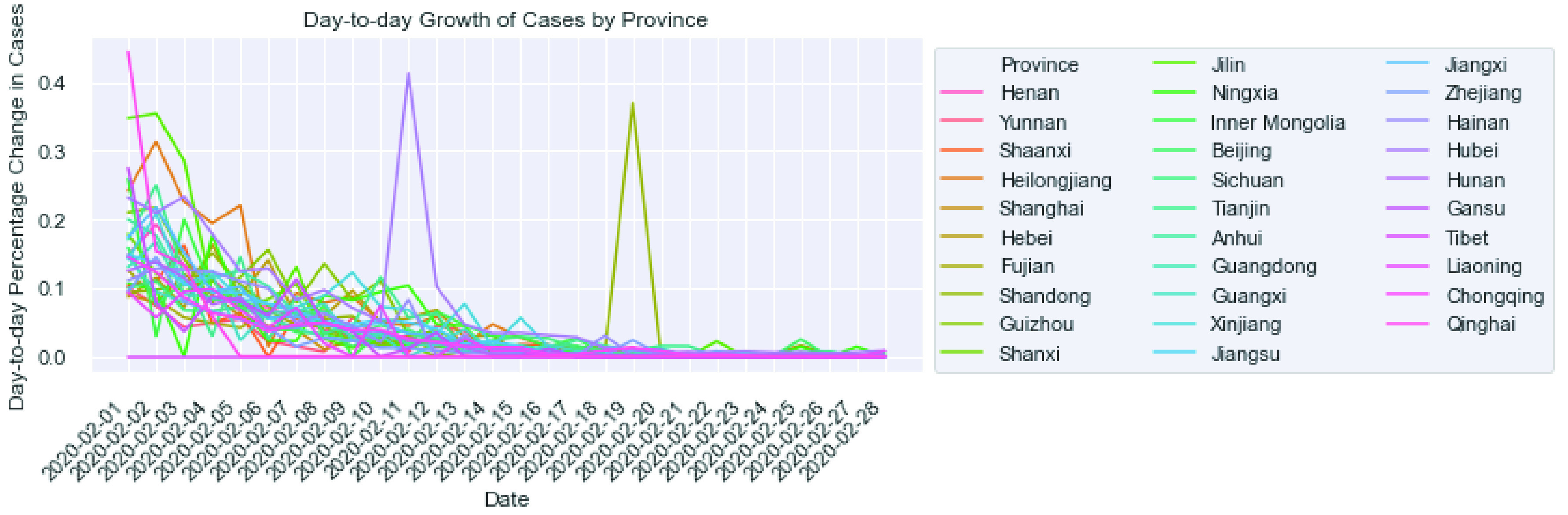
Day-to-day growth of COVID-19 confirmed cases during February 2020.

Fig. 5 shows the day-to-day percentage growth in the total number of confirmed cases in February 2020. In general, the growth rate in new cases dropped towards the end of February, except for Hubei (which modified its statistical caliber to include the individuals whose clinical evidence implies infection but formal test results had not yet come out) and another province (spotted a new cluster of infected individuals).

### B. Stage 1

Equipped with cleaned data, in the first stage, we applied the pre-trained SKEP model (available at https://github.com/baidu/Senta) on cleaned Sina Weibo tweets. In fact, the pre-trained model could accomplish three tasks (as they have been jointly optimised): (i) sentence-level sentiment classification (1 for positive and 0 for negative); (ii) aspect-sentiment prediction; and (iii) opinion extraction. The use of the aspect-sentiment module might look appealing, but the implementation was not easy in our study because the “aspect” word for COVID-19 takes various forms in Sina Weibo tweets and could even be absent from the content. Thus, we adopted a sentence-level sentiment analysis of the tweets.

**FIGURE 6.**
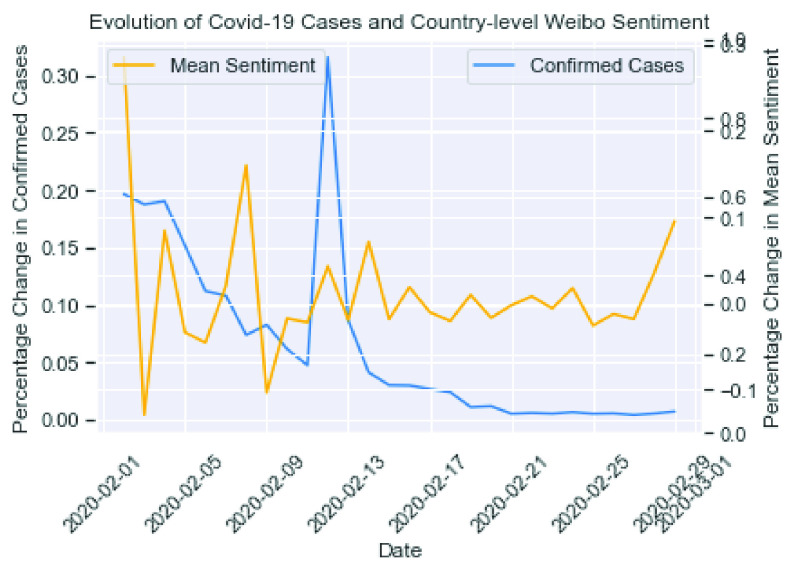
Change in cases vs. change in sentiment in February 2020.

Fig. 6 compares the daily percentage changes in country-wide confirmed cases versus the mean sentiment. As explained in the previous section, the spike in cases on February 12, 2020, was not an incorrect record due to the change in Hubei’s caliber. Overall, we observed a gradual decrease in new cases, and the change in sentiments became more stabilized towards the end of the month.

### C. Stage 2

To demystify and validate the SKEP prediction, we performed an RF to rank the importance of words appearing in the $1-gram$ vocabulary of input texts and ran an LP model to examine whether seeing the occurrence of a word would drive the sentiment classification towards 1 or 0. As shown in the figure, we implemented various cleaning steps to filter out noise and tokenised Sina Weibo tweets using the particular version of PKUSEG specialized for social media texts.

In Particular, we first applied a 5-fold stratified cross-validation to confirm that the hyperparameter set makes sense. In each fold, the ratio of 0’s to 1’s is approximately 1 to 2, and the ratio of training observations to testing is approximately 4 to 1. All three measures (precision, recall, and F1-score) in Table 2 exceed 80% on average, validating that the parameters that we chose in RF are reasonable.

**TABLE 2:** Mean Five-Fold Cross-Validation Results of Random Forest

	Precision	Recall	F1-score	Support
0	0.7843	0.6201	0.6926	22649
1	0.8285	0.9150	0.8696	45442
Weighted Avg.	0.8139	0.8169	0.8107	68091

We next used the same set of parameters but trained RF on the population of input texts. We invoked the *sklearn* package in *Python* to complete the training and automatically produced feature importance scores based on the information gain.

**FIGURE 7.**
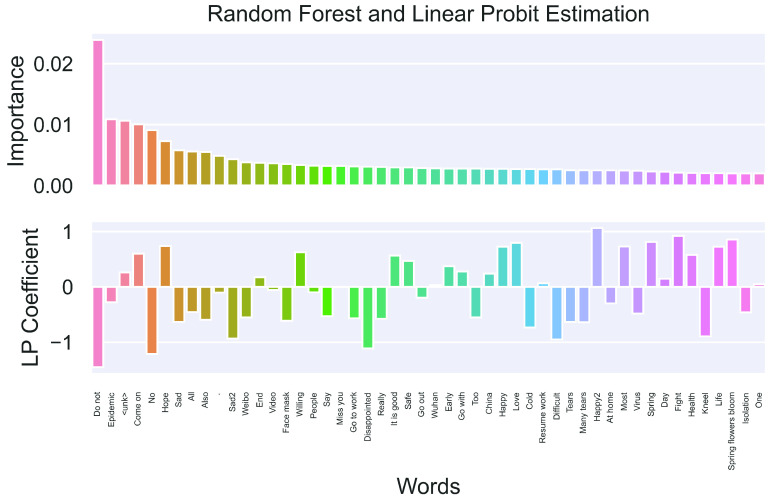
Stage 2 results: Word importance and coefficients.

The top panel in Fig. 7 visualizes the importance scores of the top 50 most important words selected by the RF. We trained the RF on the original Weibo tweets written in Chinese, and all of the words on the x-axis were translated from their Chinese counterparts. However, there are still two noisy words: “<unk>” (i.e., unknown Chinese character or symbol) and “$\cdot $,” but the remaining 48 words are all semantically meaningful. Finally, on the x-axis, we have “sad” along with “sad2” and “happy” along with “happy2” because each word is translated from the Chinese words that are almost identically express the “sad” and “happy” moods.

Among the 50 most important words, words directly related to COVID-19, such as “epidemic,” “virus,” and “mask,” are indeed considered to be influential in classifying the mood. In addition, common sentiment words, such as “sad,” “happy,” and “come on,” play a substantial part in making the prediction.

To better understand the sentiment polarity of each word in the context of COVID-19, we implemented an LP model with the tf–idf scaled occurrence of the top 50 most important words being the input and SKEP prediction as the response. The bottom panel of Fig. 7 visualizes the estimated coefficients with 0 as the horizontal reference line. A positive (negative) coefficient indicates that the focal word would drive the prediction towards 1 (0), *ceteris paribus*. For example, “come on,” “hope,” and “love” all have relatively large positive coefficients, suggesting that they strongly express a positive sentiment. Furthermore, words, such as “sad,” “disappointed,” and “tears” are all negative sentiments, indicating that tweets containing them are most likely to be pessimistic. The coefficient of “epidemic” itself is minor in magnitude, likely because this word usually occurs in a mixture of contexts. Sometimes, people express their worries with “epidemic” included, and in other cases, people also cheer each other up also with “epidemic” in the tweet.

The numerical estimates of the word importance and LP coefficients are listed in the Appendix (Fig. 9).

**FIGURE 8.**
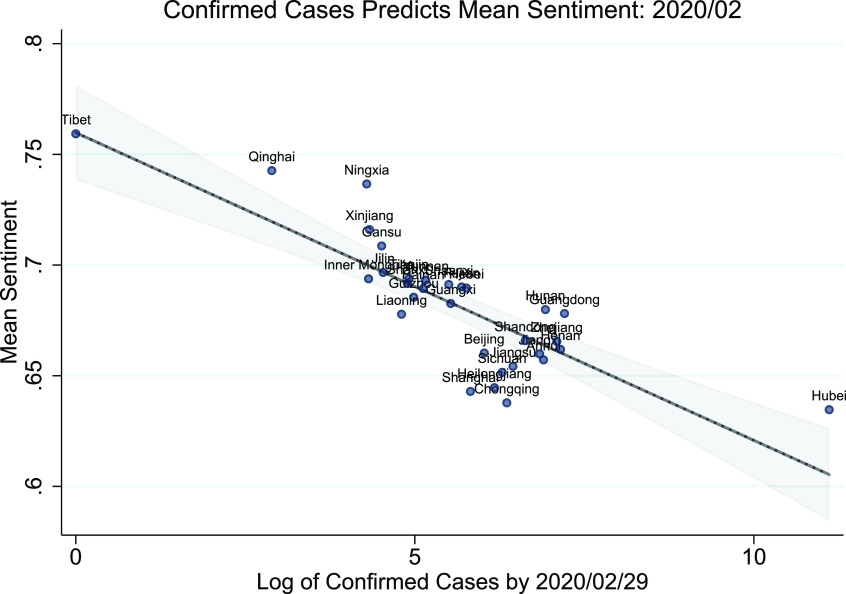
The negative linear relationship between COVID-19 severity and sentiment at the province level.

**FIGURE 9.**
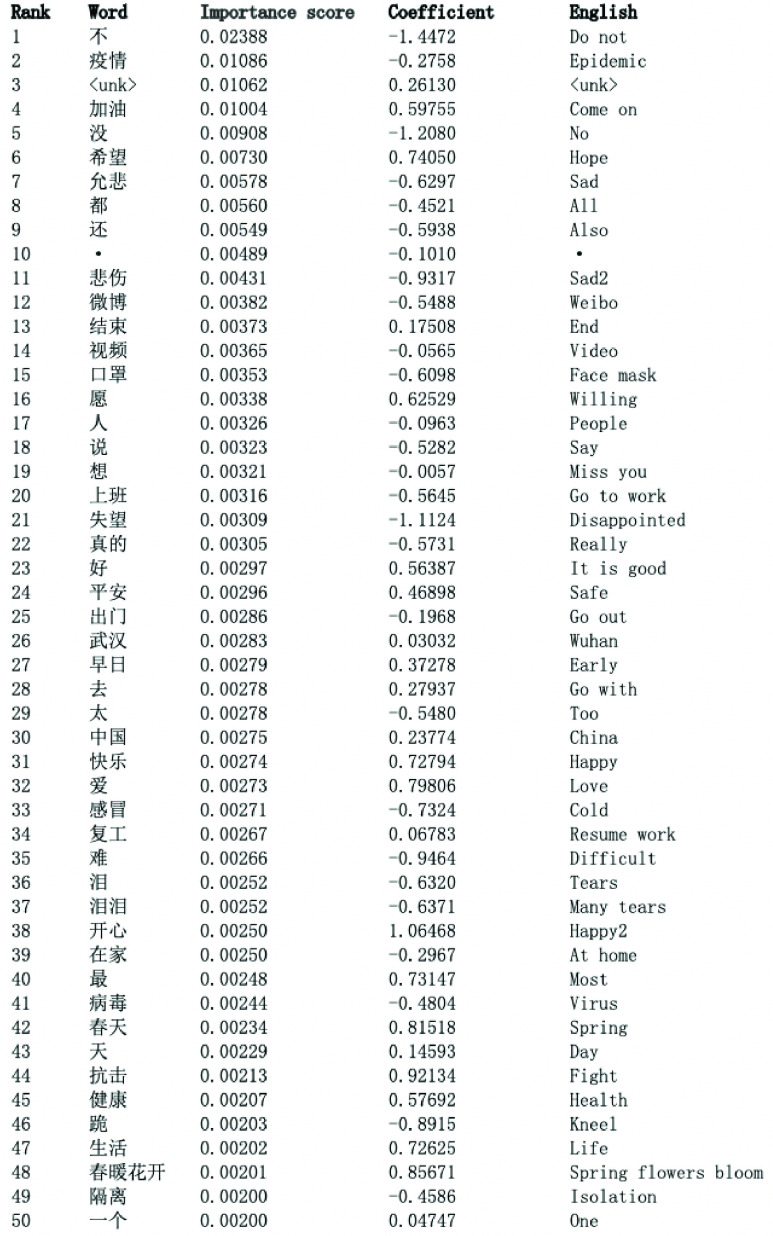
RF word importance scores and LP coefficients.

Overall, RF reasonably selects meaningful words to classify COVID-19-related sentiments, and LP also sensibly assigns the word polarity of the selected words. Because all of the trainings here are based on the Stage 1 prediction, the RF and LP results help to corroborate the validity of using the pre-trained SKEP model in analysing COVID-19-related social media texts.

### D. Stage 3

With the labels obtained in Model 1, we regressed the local sentiment responses on COVID-19 severity. On the $Y$ side, we collapse sentiment labels to the province level from February 1, 2020, to February 29, 2020, the most distressed pandemic period in China. After the aggregation, we have 31 ratios between 0 and 1, indicating the share of positive moods among all Sina Weibo tweets with location information in each province.

On the regressor side, we conducted a $\log $ transformation to render the scale of pandemic severity comparable across different provinces. Control variables were included to account for baseline provincial heterogeneity, that is, ensuring that basic geoeconomic differences across provinces would not distort the estimation of the $\log (Cases)$ coefficient. These variables include the 2018 share of the urban population, total population, nominal GDP, share of the manufacturing, and service industries in the whole economy. The data are from the 2018 China Statistical Yearbook.

Table 3 presents the estimated regression coefficients for the linear regression with two configurations: excluding and including the control variables. Standard errors are listed in the parentheses below each coefficient estimate. In either setting, the coefficient of $\log (Cases)$ is significantly negative at the 1% confidence level. The difference in magnitude (i.e., −0.014 compared to −0.0087) suggests that geoeconomic differences could explain part of the variation in the local sentiment responses to COVID-19. Remarkably, even without any controls, the $R^{2}$ of the univariate regression is 0.68, indicating that 68% of the variation in sentiment is solely driven by the pandemic.

**TABLE 3:** Local COVID-19 Severity Predicts Local Sentiment Response

	(1) Sentiment	(2) Sentiment
Log confirmed cases, end of month	−0.0140*** (0.0021)	−0.0087*** (0.0017)
2018 Urban population share		−0.00026 (0.00053)
Log 2018 population (million)		−0.0069 (0.013)
Log 2018 nominal GDP		−0.0057 (0.014)
2018 share of manufacturing industry		0.0012 (0.00072)
2018 share of service industry		0.00012 (0.00081)
Controls	No	Yes
$R^{2}$	0.68	0.80
Observations	31	31

Standard errors in parentheses

* $p < 0.1$, ** $p < 0.05$, *** $p < 0.01$

Fig. 8 shows the estimated linear trend of the univariate linear model compared to the true value. The graph shows that almost all 31 provinces are closely aligned along the estimated line. Hubei, the centre of the COVID-19 pandemic in China, is slightly off the line, likely because the government implemented assorted measures to psychologically boost the confidence of local citizens.

In short, the linear regression validates the hypothesis that areas that suffered more during the pandemic would have a smaller extent of optimism on social media.

## IV. Conclusion

In this study, we applied the most state-of-the-art pre-trained sentiment classification model, SKEP, to classify COVID-19-related social media tweets in China. Because SKEP itself does not provide a feature explanation, we implemented an RF followed by an LP model with SKEP prediction as the response variable, and we confirmed the SKEP classification. The visualization of the RF and LP models shows that COVID-19-related words and common sentiment words play a substantial role in driving the prediction. Finally, we aggregated sentiment classification from the tweet level to the province level for all COVID-19-related tweets with recognizable GPS information. Univariate and multivariate linear regression results corroborate the hypothesis that more severely affected regions tend to have a greater share of pessimistic moods. In fact, comfirmed COVID-19 cases alone could explain approximately 68% of the variation in sentiment across provinces.

Technically, to further improve the explanatory power of our RF and LP models, one could design and perform even finer text preprocessing to filter out additional noise. Future works could be devoted to improving the pre-trained SKEP model so that it simultaneously makes predictions and explains feature roles. In terms of drawing a broader socioeconomic conclusion, one could also apply SKEP with an RF and LP model on COVID-19-related tweets using data from other regions around the world.

## Appendix

In Fig. 9, we tabulated the top 50 most important Chinese words (the 2nd column) chosen by the RF and their importance scores reported by *sklearn*. The last column contains their English translations. In the 3rd column, we listed their LP coefficients. Here, a positive (negative) coefficient indicates that the occurrence of this word will drive the prediction towards 1 (0), *ceteris paribus*.
